# Severe psychosis due to Cushing’s syndrome in a patient with a carcinoid tumour in the lung: a case report and review of the current management

**DOI:** 10.1186/s12957-015-0571-0

**Published:** 2015-04-30

**Authors:** Mohamad Baba, Debamalya Ray

**Affiliations:** Department of Cardiothoracic Surgery, The University Hospital of North Staffordshire NHS Trust, Newcastle Road, Stoke-on-Trent, ST4 6QG England UK

**Keywords:** Cushing’s syndrome, Carcinoid tumour, Ectopic ACTH secretion, Hypercortisolaemia, Psychosis, Surgical resection of tumour

## Abstract

Severe psychosis in patients with Cushing’s syndrome is a rare occurrence and can be extremely resistant to medical therapy. We describe a case of a 51-year-old Afro-Caribbean female patient, with refractory severe hypertension (initially resistant to polypharmacy) and gradual development of severe psychosis secondary to ectopic Cushing’s syndrome, who was subsequently diagnosed to have a carcinoid tumour in her lung. Her psychotic episodes - secondary to hypercortisolism and initially refractory to the medical therapy - subsided only after the resection of the carcinoid tumour in her right lower pulmonary lobe. Early localization and appropriate surgical resection of the ectopic ACTH-secreting tumour can be of immense value to the successful alleviation of the psychotic episodes of the patients with ectopic Cushing’s syndrome.

## Background

Cushing’s syndrome is characterised by a distinct set of signs and symptoms associated with the exposure to increased concentration of cortisol hormone (exogenous or endogenous). Exogenous administration of glucocorticoid (iatrogenic) is the most common cause of Cushing’s syndrome, and the pituitary adenomas account for 80% of endogenous Cushing’s syndrome [1]. Ectopic ACTH secretion occurs in 15% to 20% of patients with ACTH-dependent Cushing’s syndrome [2] with the lung being the origin of over 45% of ACTH-producing tumours (ectopic), followed by thymus (11%) and pancreas (8%) [3,4]. Although hypertension and hypokalaemia are common findings in patients with Cushing’s syndrome at the hospital, altered mental status can be the first manifestation perceived by family members of those patients. Enhancement of dopaminergic activity by the glucocorticoids may explain the development of psychosis in cortisolaemia [5] and hence explain the resolution of the psychiatric symptoms once cortisol levels in blood are normalised. Medical treatment can be useful in decreasing the elevated blood cortisol levels [6,7] and in improving psychotic symptoms [8] to a certain degree. That the surgery is the first choice treatment for ectopic ACTH-secreting tumours (intra-thoracic neoplasms being the majority of the ectopic sources) is a widely accepted standard of care in the management of those patients [2].

## Case presentation

**Figure 1:**
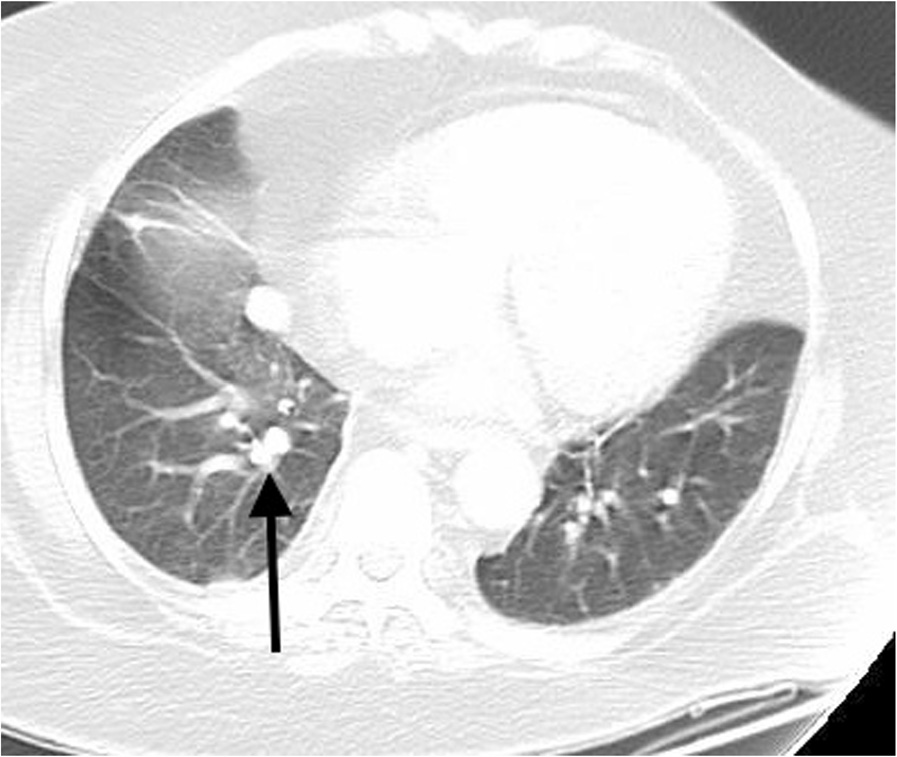
Dense nodule in the right lower lobe measuring 17 × 13 mm.

A 51-year-old Afro-Caribbean lady with a known background history of primary hyperparathyroidism, type 2 diabetes, hypertension, gradually increasing episodes of confusion over the last 2 weeks, and left leg cellulitis and without any previous documented history of any psychiatric disorder was admitted to the “Acute Medical Unit”. Subsequently, she required antibiotic therapy for her leg cellulitis. During her stay at the hospital, she developed hypokalaemia (3.7 mmol/L) associated with severe hypertension (220/120 mmHg) resistant to seven different oral antihypertensive medications. Blood test at that point in time demonstrated elevated levels of ACTH (250.9 ng/L) and cortisol (1058 nmol/L). On physical examination, the patient was noted to be centrally obese in association with the other Cushingoid features (wasting of the limb muscles, round facies, prominent eyes, extensive purple striae on her abdomen, and multiple skin lesions with hyperpigmentation). The patient required isolation in a side room as she was very confused, agitated, paranoid, and physically aggressive towards the nursing staff members. She was reviewed by the psychiatrists who suggested an initial therapy with haloperidol, followed by the commencement of a regular daily dose of 10 mg of olanzapine orally, and subsequent treatment with intravenous clonidine infusion. However, those measures failed to achieve the desired control over the episodes of her severe psychosis at the hospital. She underwent low-dose and high-dose dexamethasone suppression tests which failed to suppress her elevated blood cortisol levels thereby confirming the possibility of an ectopic source of ACTH production. Subsequently she was treated with oral ketoconazole 400 mg three times daily and intravenous etomidate 0.05 mg/kg/hr which only managed to reduce the elevated blood cortisol levels to a certain extent over the course of the next few days. An MRI scan of the brain revealed a normal hypophysis. The CT scan of her thorax (Figure 1) demonstrated a dense lesion situated in the right lower pulmonary lobe and gradually enlarging bilateral adrenal glands (as compared to the findings on the previous CT scan). However, octreotide scan did not reveal any octreotide avid lesion inside the thorax, abdomen, or pelvis. Subsequently, she underwent surgical resection of her lung lesion, and the histology examination of the resected pulmonary lesion revealed a typical carcinoid tumour. Blood test repeated on the second post-operative day showed a significant reduction of blood cortisol level to 97 nmol/L, prior to the stabilisation of the blood cortisol concentration at around 300 nmol/L over the next few days. Post-operatively, the patient was commenced on oral hydrocortisone in order to avoid the rebound effect of sudden reduction in blood cortisol level. Within 1 week following her pulmonary resection, she started registering a significant improvement in her psychotic symptoms and cognitive function. The requirement of her antihypertensive medications also decreased with better control of her hypertension due to less potentiation of epinephrine’s vasoconstrictive effect by reduced level of cortisol in the blood stream. Subsequently, the patient was transferred to a peripheral hospital in a stable condition for further rehabilitation.

## Conclusions

Cushing’s syndrome can be diagnosed clinically on the basis of a thorough physical examination. However, further investigations are required in order to confirm the diagnosis and to locate the source of excessive ACTH/cortisol secretion. Localization of the source of elevated blood ACTH level can be troublesome with some pituitary tumours being less than 2 mm in size and difficult to detect using MRI or CT imaging. Using a modern multi-detector high-resolution CT scan capable of acquiring images 16 to 24 slices per second and 2.5 mm slices from the lung apex to the iliac crests is recommended [9]. In the case described above, administration of dexamethasone suppression test and demonstration of a normal hypophysis on the MRI scan of the brain were enough to exclude the presence of Cushing’s disease as a cause of hypercortisolaemia. The pulmonary lesion detected by the CT scan of her thorax (Figure 1) was resected surgically and confirmed to be a carcinoid tumour of the lung on histology examination. Bronchial carcinoids typically tend to have a prolonged history and slow onset of symptoms (1 to 84 months, median 23.6 months) [3]. Bronchial carcinoids as small as 0.5 mm may produce florid Cushing’s syndrome [10]. In most cases, patients with bronchial carcinoids do not tend to develop any prominent respiratory signs or symptoms attributable to the lung disease making those tumours hard to detect clinically [11].

Surgical resection of the tumour remains the optimal treatment for ectopic ACTH-related Cushing’s syndrome. Medical therapy plays a role in those patients who are not deemed suitable for or unwilling to undergo surgery. Several drugs have been found to inhibit cortisol synthesis (ketoconazole, metyrapone, etc.) with limited efficacy [12]. Ketoconazole has been shown to be effective in more than 50% of patients with ectopic ACTH syndrome [13] and is associated with many side effects. Etomidate infusion is an alternative for patients unable to take oral medications and is better tolerated than ketoconazole. Mifepristone - a glucocorticoid type II receptor antagonist - does not interfere with the normal cortisol homeostasis type I receptor transmission and may be especially useful for treating the cognitive effects of Cushing’s syndrome [12].

Patients with identifiable source of ectopic ACTH production should undergo surgical resection of the concerned tumour. Surgery may be curative in more than 80% of bronchial carcinoids. After correction of hypercortisolism, resolution of psychiatric symptoms is variable. The majority of patients will improve in the first few weeks [14,15]; however, 36% of patients will still be symptomatic after 12 months [15]. Despite using modern techniques, a source of ectopic ACTH production may not be detected in almost 12% of patients. In such cases with occult ACTH-secreting tumours, bilateral adrenalectomy, preferably performed laparoscopically, constitutes a therapeutic option with low morbidity and mortality in experienced hands. However, those patients will require long-term glucocorticoid and mineralocorticoid replacement therapy for obvious reasons [11]. Patients with primary intra-thoracic tumours, especially bronchial carcinoid, are more likely to be cured than patients with primary intra-abdominal tumours [16]. Failure of surgical resection is mainly due to a primary tumour that has already metastasized.

## Consent

Written informed consent was obtained from the patient for publication of this case report and any accompanying images. A copy of the written consent is available for review by the Editor-in-Chief of this journal.

## Abbreviations

ACTH: Adrenocorticotropic hormone
CT: Computed tomography
MRI: Magnetic resonance imaging
